# Individual-level characteristics and geospatial factors associated with cervical cancer screening participation in Alberta, Canada: a population-based cross-sectional study

**DOI:** 10.1186/s12889-025-23898-4

**Published:** 2025-08-15

**Authors:** Jessica Law, Geneviève Jessiman-Perreault, Amanda Alberga Machado, Linan Xu, Bonnie Chiang, Huiming Yang, Lisa Allen Scott, Rizwan Shahid, Curtis Mabilangan, Alvin Li, Kamala Adhikari, Gary Teare

**Affiliations:** 1 Provincial Population and Public Health, Alberta Health Services, Holy Cross Centre, 2210 2 St SW, Calgary, AB T2S 3C3 Canada; 2 Screening Programs, Provincial Population and Public Health, Alberta Health Services, 10101 Southport Rd SW, Calgary, AB T2W 3N2 Canada; 3 Department of Medicine, Cumming School of Medicine, University of Calgary, 3330 Hospital Drive NW, Calgary, AB T2N 4N1 Canada; 4 Health Services Research and Innovation - Primary Health Care, Alberta Health Services, Southport Tower, 10301 Southport Lane SW, Calgary, AB T2W 1S7 Canada; 5 Department of Community Health Sciences, Cumming School of Medicine, University of Calgary, 3330 Hospital Drive NW, Calgary, AB T2N 4N1 Canada

**Keywords:** (3–10): cervical cancer, Cancer screening, Epidemiology, Spatial analysis, Hot-spot

## Abstract

**Background:**

Cervical cancer is the fourth most common cancer in women worldwide. Effective primary prevention with human papillomavirus vaccination and secondary prevention with screening can prevent most cervical cancer cases. Cervical cancer screening uptake varies among women in underserved populations. Research that adds to the understanding of the individual and geographic area-level characteristics of women and their screening status is valuable for public health intervention planning. This study aimed to identify these characteristics related to cervical cancer screening status.

**Methods:**

The study population included women between the ages of 28 to 69 years in Alberta. Data was extracted from administrative health data sources and linked to the Alberta Cervical Cancer Screening Program database to determine screening status. Descriptive bivariate analysis was conducted to describe variations in cervical cancer screening statuses and individual-level sociodemographic, health system factors, and geographic characteristics. Multinomial logistic regression analysis was conducted to investigate the relationship between these characteristics and screening participation. Geospatial analyses including heat maps were used to visualize variation in screening participation across the province. Getis-Ord Gi* hot-spot analysis was used to determine the location and magnitude of spatial autocorrelation.

**Results:**

The study included 933,965 eligible women. Compared with those who are currently up-to-date for screening, those who have no record of screening tend to be older (OR: 3.63; 95% CI: 3.57 to 3.70), reside in the South Zone (OR: 1.51; 95% CI: 1.47 to 1.55), were health system non-users (OR: 2.95: 95% CI: 2.86 to 3.04), did not see a general practitioner (OR: 13.86; 95% CI: 13.32 to 14.43), or had no usual provider of care (OR: 3.227; 95% CI: 3.141 to 3.315). There are statistically significant hot spots of women who are overdue or have no record of cervical cancer screening in the North, Central, and Calgary Zones.

**Conclusions:**

This study found that cervical cancer screening participation varied across geographical, health system and sociodemographic characteristics and identified clusters of regions with higher proportions of women who are under-screened in Alberta, Canada. Overall, these findings will help inform the design of interventions that aims to improve cervical cancer screening participation among underserved groups.

**Supplementary Information:**

The online version contains supplementary material available at 10.1186/s12889-025-23898-4.

## Background

Cervical cancer is the fourth most common cancer in women worldwide [1], but it is highly preventable and curable [2]. Effective primary prevention with human papillomavirus (HPV) vaccination and secondary prevention with cervical cancer screening, such as Papanicolaou (Pap) tests, and treating precancerous lesions can prevent most cervical cancer cases [2]. In alignment with the World Health Organization’s (WHO) Global Strategy for cervical cancer elimination [3], Canada is aiming to eliminate cervical cancer by 2040 through improvement in HPV immunization rates, implementing HPV primary screening, and improving follow-up of abnormal screening results [2].

In 2017, cervical screening participation for women 21 to 69 years of age was 76.6% in Canada, below the target of 90% [4]. Despite having universal health care and organized provincial screening programs, variations in cancer screening rates exist by socioeconomic status (SES), geography, access to care, minority groups and immigrant status [5]. These disparities are expected to increase due to disruptions in the provision and uptake of screening services due to the COVID-19 pandemic [6]. There is therefore a need to implement effective interventions to restore screening rates to pre-pandemic levels and improve rates to reach the provincial screening target for Alberta [7] by understanding current barriers to cervical cancer screening. Socioeconomic and financial factors including indirect costs like childcare and taking time off work are barriers to cervical cancer screening [8–11] resulting in lower screening participation rates among people with lower SES compared to those with higher SES [12]. These factors have also been identified as important determinants of screening rates internationally [13].

Factors affecting access to health services including lack of a family physician, or having no source of regular care have been identified as barriers to screening [5, 10, 11]. Additionally, geographic disparities in cervical cancer screening also exist. For example, in the United States, women living in rural areas were less likely to complete cervical cancer screening with access to consistent health care being a potential contributor to the lower participation rate [14]. Results from a geospatial study done in Calgary, Alberta found a relationship of material and social deprivation with cervical screening outcomes, which resulted in geographic clusters of lower cervical cancer screening participation [15]. Therefore, there is evidence of the influence of socioeconomic, healthcare, and geographic factors on cervical cancer screening participation, but few studies examine these factors simultaneously.

Thus, the objective of this study is to identify and describe the geographical, health system, and sociodemographic characteristics related to eligible individual’s cervical cancer screening statuses. This study was conducted by Alberta Health Services (AHS), the provincial healthcare authority, to support decision-making on the development of interventions to increase cervical cancer screening in Alberta by targeting important geographic, health system and sociodemographic barriers. Specifically, we aim:

1. To examine differences and determine the association of socioeconomic, health system, and geographic characteristics with cervical cancer screening status (i.e. women who have no record of screening (NRS), who are overdue, and currently up-to-date (CUTD) for cervical cancer screening).
2. To describe variation in cervical cancer screening status across geographies and identify clusters of regions with higher proportion of women with no record or are overdue for cervical cancer screening in Alberta, Canada.

## Methods

### Study design and population

This is a population-based cross-sectional study where the cervical cancer screening status of women in Alberta was assessed as of December 31, 2019 (index date). In Alberta, it is recommended that screening occurs once every 3 years among asymptomatic women, aged 25 to 69 who are at average risk of cervical cancer (i.e., sexually active, asymptomatic women) [16]. The Alberta Cervical Cancer Screening Program (ACCSP) supports residents of Alberta in accessing cancer screening across the province. The ACCSP supports Albertans in accessing screening through direct communication in the form of invitations and reminders, as well as through the coordination of health services via healthcare providers. Invitations are sent when women turn 25 and have no record of cervical cancer screening. Reminders are sent when women are due for their next screen or follow up tests. The ACCSP works with healthcare providers to ensure quality services and provide standard screening information.

Cervical cancer screening is done with a Pap test, which can detect precancerous lesions early and allows for earlier treatment and management [17]. If results of the Pap test are abnormal, follow-up with the healthcare provider is recommended and further testing may be required. While it is recommended that women start cervical cancer screening at age 25, the study includes women aged 28 to 69 years of age. The starting age of 28 was selected to allow women the opportunity to get screened within 3 years from when they first became eligible, in accordance with current cervical cancer screening guidelines in Alberta. This study has received research ethics approval from the Health Research Ethics Board of Alberta (HREBA.CC-20-0425).

### Inclusion and exclusion criteria

**Fig. 1:**
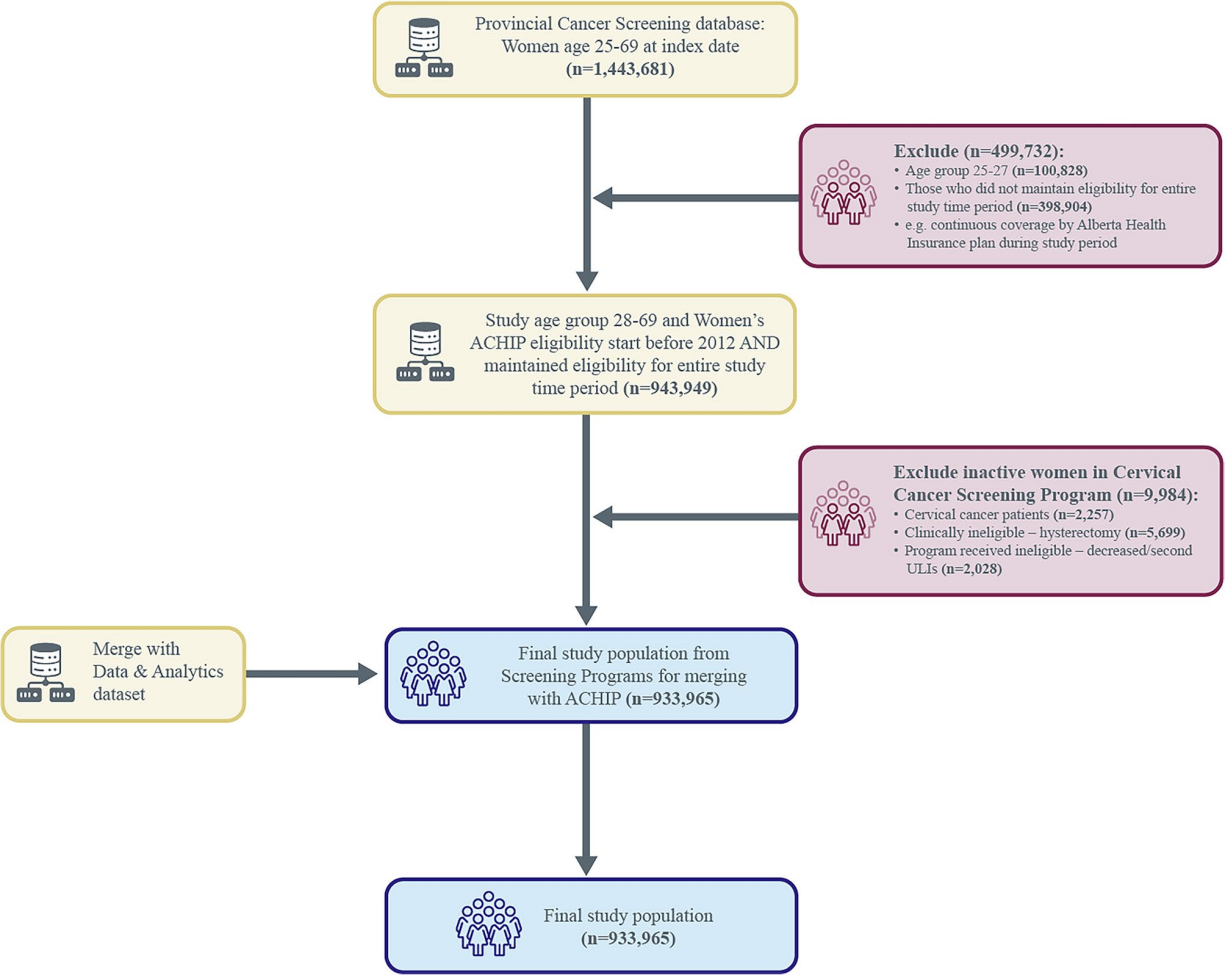
Study dataset creation flow chart

The starting point to abstract the population data comprised women who had a valid unique Personal Healthcare Number (PHN) along with their date of birth. Women included in the sample were those alive and covered by Alberta Health Care Insurance Plan (AHCIP) from January 1, 2012 to December 31, 2019, who were between the ages of 28 and 69 years on the study index date (i.e., December 31, 2019). January 1, 2012, is the starting date from which the population of Alberta received cervical cancer screening coverage from the ACCSP, hence, no data prior to 2012 exists. Anyone who may have been screened prior to this period was classified within the “no record of screening” category, along with those who have no record of screening during the study period. Individuals were excluded from the study sample for: (1) having an “inactive” ACCSP program status, (2) a history of cervical cancer diagnosis or hysterectomy, (3) if they had actively opted out from Alberta Screening Programs’ tracking of their data, or (4) if they hade missing data on date of birth, sex, postal code; or invalid PHN. After exclusions, the final dataset included data from 933,965 women (see Fig. 1 for study cohort creation).

### Data sources and linkage

Multiple administrative datasets from AHS were linked using the woman’s PHN or Unique Lifetime Identifier (ULI) to create the dataset for this study. The provincial ACCSP database includes key information on cervical cancer screening status, age, postal code, time overdue for screening and history of cancer at the time of the index date to create the population of those eligible to be screened and ascertains screening participation data. First, these data were linked to the Alberta Health Person Directory within AHCIP, which contains population demographics for all persons covered for basic medical and hospital insurance during a given fiscal year. Data for women were selected based on age and eligible status for this study. Two administrative sources of health care utilization data were linked to determine co-morbidities: the Physician Claims dataset and the National Ambulatory Care Reporting System (NACRS). The Physicians Claims dataset contains the date of service and related diagnostic and treatment information submitted for fee-for-service billing. The number of primary care visits and measures of continuity of care were extracted from the Physician Claims dataset, which was linked to the individual through their PHN. The NACRS contains health administrative, demographic, and clinical and service specific data for all hospital- and community-based ambulatory care including day surgery, emergency department, outpatient, specialty care, and community clinic visits. Additionally, the 2016 Pampalon Index Dataset, a small area–based composite index that uses census data at the Dissemination Area (DA) level, was linked and provided sociodemographic data of material and social deprivation [18].

Finally, geographic data like postal code, urban/rural continuum and Alberta Health zone were extracted from Alberta Health Postal Code Translator File (PCTF). On the index date (December 31 st, 2019), the postal codes were derived based on health insurance data instead of screening administrative data. This is because women classified as no record of screening did not have any examinations recorded in the screening database, and as a result, no corresponding postal codes were available for them. Individual postal code was geocoded using the 2016 Alberta Health PCTF. The distance from each individual’s postal code to the closest primary care clinic was calculated using data from the College of Physicians and Surgeons of Alberta and the 2021 AHS DMTI Route Logistics Road New File. The dataset listing all primary care clinics in Alberta which offer cervical cancer screening was linked to driving time and distance from each postal code of women in Alberta to all primary care clinics in Alberta. The screening and administrative database and health insurance data are well integrated comprehensive databases that were directly linked by PHN. No issues with data linkage were detected.

### Outcome measures

The primary outcome of interest was a three-level screening participation status variable based on whether they have a record of Pap test within 42 months prior to the index date. The 42-month time interval allows women the time to get screened and accounts for health system booking and access logistics. This is aligned with ACCSP participation rate calculation definition as adopted from the Canadian Partnership Against Cancer’s key performance metric. Those who were considered currently up to date (CUTD) had a Pap test or record of colposcopy anytime within the last 42 months of the index date. Those who were Overdue had a pap test greater than 42 months of the index date. Those who had no record of screening (NRS) were those not considered CUTD or overdue for cervical cancer screening and had no record of screening in the ACCSP database during the study time frame.

### Sociodemographic measures

Variables for inclusion in this analysis were selected a priori based on review of scientific literature to identify factors that impact cervical cancer screening participation and outcomes, relevance to research questions and data availability. Results from a geospatial study done in Calgary, Alberta found a relationship between material and social deprivation and cervical screening outcomes where there were geographic clusters of lower cervical cancer screening participation [15].

Sociodemographic measures that were examined in this study were age, material deprivation, and social deprivation. Social and material deprivation were measured using the Pampalon Index. The social deprivation index measures the deprivation of relationships among women in the family and community while the material deprivation index measures the deprivation of wealth, goods and convenience [18].

### Geographic measures

Cervical cancer screening is also impacted by geographic disparities. Studies have found that those living in rural areas were less likely to complete cervical cancer screening, noting that access to consistent health care was a potential contributor to the lower participation rate [11, 14]. Geographic indicators of interest include local geographical area (LGA), urban/rural continuum, and Alberta Health zone. These three measures were developed in 2010 by AHS and Alberta Health to provide standard geographic measures for the province to aid in the surveillance, monitoring, planning, and reporting of health outcomes and service use across the province. These measures were developed based on the DA from Statistics Canada and undergoes regular revisions [19].

**Fig. 2:**
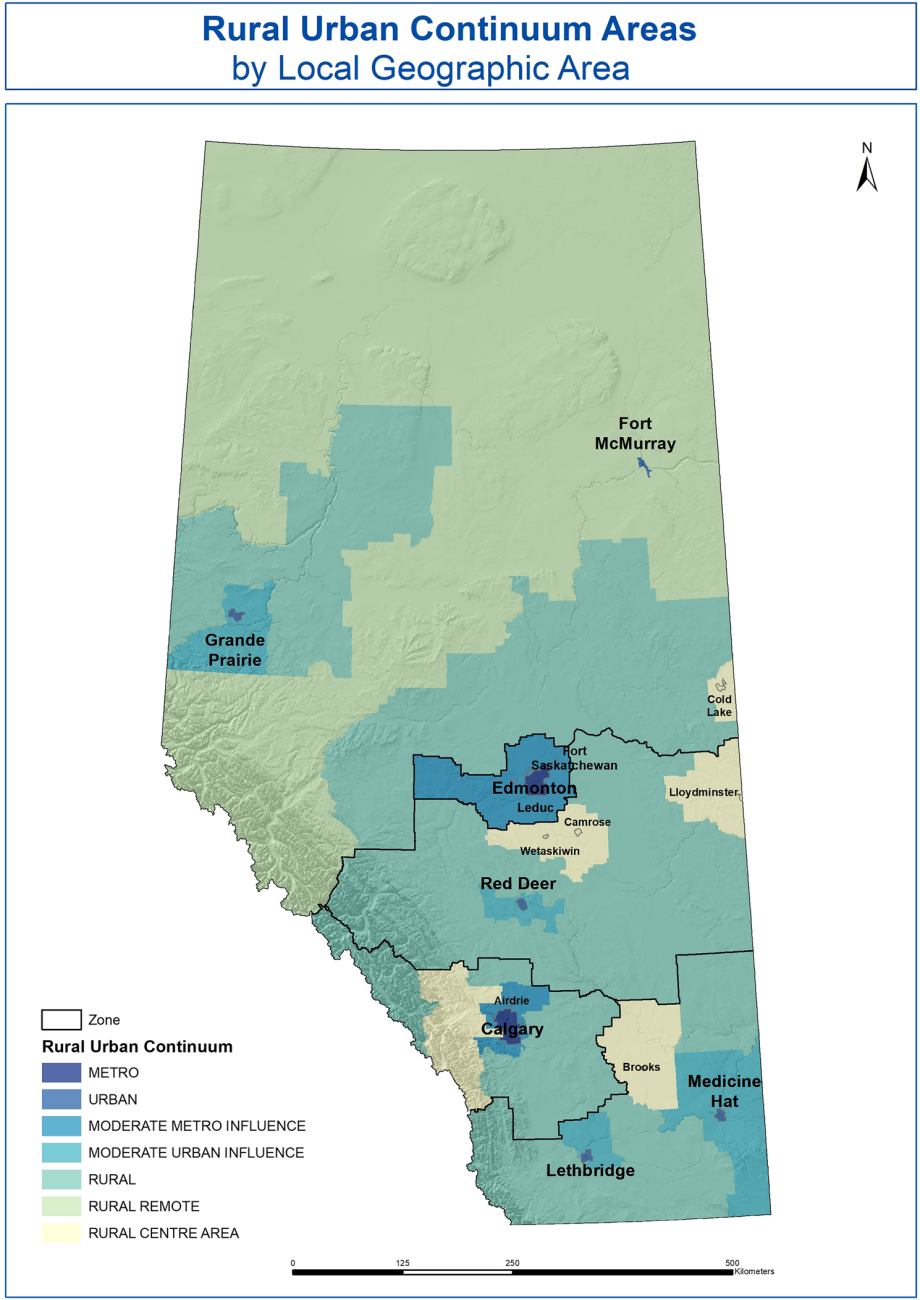
Rural Urban Continuum Areas by LGA

LGA was generated based on the individual’s postal code. In Alberta there are 132 LGAs of varying sizes across fives health zones (North Zone, Edmonton Zone, Central Zone, Calgary Zone, and South Zone). The rural-urban continuum includes seven types of areas and are based on the aggregation of LGAs based on factors including population density and distance from urban or rural centres [19]. Based on the rural-urban continuum, the areas were grouped into either urban (metro centres, metro influenced area, urban, and moderate urban influenced) or rural (rural centre areas, rural, and rural remote) areas (see Fig. 2).

### Patient health and health system measures

Factors that impede the interaction with the healthcare system like lack of provider contact, logistical concerns like having no source of regular care, and geographic access issues like those who have to travel long distances to receive screening have been identified as barriers to screening [11]. Therefore, health system measures that were examined in this study were general practitioner (GP) visits, continuity of care, Canadian Institute for Health Information (CIHI) Population Grouping Methodology (POP Grouper), and distance to the closest primary care clinic. Based on the number and timing of primary care visits, a binary variable (yes, no) was generated to indicate whether or not the individual had attended a primary care visit in the three years prior to index date.

Usual provider of care (UPC) is an established measure used to assess continuity of care over a period of time [20–22]. In the study, the UPC score is derived by utilizing all community GP or family physician visits in the 3 years prior to study index date and assigned into levels of no UPC, low UPC, moderate UPC, or high UPC among those who had a usual provider of care. A score was determined for each individual in the dataset. The practitioner with the most visits during that period is considered as the ‘regular’ physician and the score is determined by the number of visits with the regular physician in the 3 years prior to study index date divided by the total number of visits with any community GP in the same period. Patients with less than 3 visits to their regular physician were classified as having no UPC. This includes those who do not have a GP and those who saw different GPs over a 3-year period thus did not have a regular physician. Those with low continuity score between 0 and 0.4, medium between 0.4 and 0.7, and high between 0.7 and 1. Multiple billing records on the same day were counted as a single visit.

The CIHI POP grouper summarizes health care utilization by placing individuals into health risk groups (health system users and non-users) based on history of health services received using data from inpatient, emergency department ambulatory, and physician claims. The health risk groups provide broad information about the type (e.g., acute, chronic, cancer) and severity (e.g., moderate, major) of the health profile group.

### Data analysis

Descriptive bivariate analysis was conducted to describe variation in individual-level sociodemographic, health system factors, and geographic characteristics by cervical cancer screening status using counts and percentages. Correlations between covariates were checked using chi-square and spearman rank-order correlation tests, where appropriate to determine the inclusion in the logistic regression analysis. Multinomial logistic regression analysis was conducted to investigate characteristics associated with having NRS of cervical cancer screening and being overdue for cervical cancer screening compared with those who are CUTD with cervical cancer screening by estimating the odds ratios (ORs) and using CUTD as the reference group. Adjusted odds ratios, where all study covariates were adjusted for, and 95% confidence intervals (CI) are reported. To adjust for multiple comparisons, we applied the Bonferroni correction as a post-hoc procedure. The multinomial logistic regression included an outcome variable with 3 categories (2 comparisons), and 9 predictor variables with multiple categories for each variable. There were 2 × 25 comparisons for a total of 50. The overall alpha level of 0.05 was divided by the number of comparisons to achieve the new Bonferroni-corrected p-value [23]. The Bonferroni-corrected alpha level (statistical significance) for the multinominal logistic regression was 0.001 (α/50 = 0.05/50 = 0.001). Multicollinearity was assessed using a correlation matrix and Variance Inflation Factor (VIF). Model goodness-of-fit was assessed using the Hosmer–Lemeshow test, which evaluates the agreement between observed and predicted outcomes, and the Nagelkerke pseudo R², which indicates the proportion of variance explained by the model. No major collinearity issues were found using a threshold of VIF < 2.5 [24]. All analyses were conducted using SAS Studio release 3.81, Windows version (Enterprise Edition).

### Spatial Analysis

Individual postal code was geocoded using the PCTF. We aggregated estimates at the LGA for percent of women overdue for and with no record of cervical cancer screening. We chose LGA as the geographic unit for this spatial analysis, as until 2020, LGAs were the lowest geographic area that AHS and Alberta Health used as official geographies for which healthcare is distributed [25]. These LGAs provide sufficient sample size to allow for accurate and precise estimates and this geographic unit has been used in other geospatial studies of health outcomes in Alberta [26–28].

First, heat maps were generated to visualize the percent of women overdue for and with no record of cervical cancer screening by LGA in Alberta (Tableau Desktop Version 2021.2.1). Next, Global Moran’s I was used to detect the presence and intensity of global spatial autocorrelation of women with NRS and women overdue for cervical cancer screening. Global Moran’s I, as implied by its name, reveals spatial autocorrelation for the entire study area. It does not assess specific regions to pinpoint the existence of spatial autocorrelation at the local level [29]. P-values less than 0.05 means we can reject the null hypothesis of complete spatial randomness and accept that spatial autocorrelation and clustering exists [29]. To discern the local-scale spatial autocorrelation clusters, the Local Indicator for Spatial Autocorrelation (LISA) was conducted [30].

Next, to detect where spatial clustering is occurring, we used local Getis-Ord Gi* hot-spot analysis, with 499 permutations, to determine the location and magnitude of spatial autocorrelation under the null hypothesis stating that the associated values of no record of or overdue for cervical cancer screening follow complete random distribution across Alberta [31]. The hot-spot analysis provides statistically significant clusters of high values, called hot spots and clusters of low values, called cold spots. We used optimized hot-spot analysis that determined the optimal fixed distance threshold and nearest neighbors as the function to conceptualized space for our hot-spot analysis. For both outcomes, the threshold distance is set at approximately 122 km, and each LGA has on average 6 nearest neighbours. We found less than 45% LGAs had fewer than 8 neighbours. Statistically significant hot and cold spots were selected using a false discovery rate (FDR) that corrects for multiple tests and spatial dependence among LGAs by adjusting p-value to avoid false identification of the significant hot or cold spots [32]. All spatial analysis was conducted using ArcGIS Pro 2.6.3 [33].

## Results

Table 1 presents the percent distribution of study variables across cervical cancer screening status. Overall, 62% of Albertan women aged 28 to 69 years old in 2019 were CUTD on their cervical cancer screening, 17% were overdue for cervical cancer screening and 21% had NRS of cervical cancer screening. Results from Table 1 demonstrate that those with NRS of cervical cancer screening tend to be older than those overdue for cervical cancer screening and those who are CUTD for cervical cancer screening. Those who are overdue (42.1%) or have NRS (45.1%) tend to be most material deprived (i.e., within categories 4–5 according to material deprivation). A similar trend is seen according to social deprivation though to a lesser extent. There is a higher proportion of those who are overdue (22.2%) or have NRS (24.7%) who live in rural areas compared with those who are CUTD (18.3%). There is a slightly higher proportion of people with NRS residing in the South (8.3%), North (12.8%), and Central (13.5%) zones compared to CUTD screeners (6.7%, 9.4%, and 10.3%, respectively). However, the proportion of those who have visited a family doctor in the 3 years prior to index is much higher amongst those CUTD (99.2%) compared with those overdue (85.5%), and those with no record of screening (66.0%). Moreover, a higher percent of those who are overdue for cervical cancer screening (23.3%) and those with NRS (40%) tend to be non-users of the health system compared with those CUTD (5.4%).

**Table 1:** Characteristics of women who are currently up-to-date, overdue and no record of cervical cancer screening (*n* = 933,965)

Characteristic	Currently Up-to-date (*n* = 575,710, 61.6%)	Overdue (*n* = 158,494, 17.0%)	No Record of Screening (*n* = 199,761, 21.4%)	*P*-value
Age				< 0.0001
28–39	163,653 (28.4)	44,817 (28.3)	42,466 (21.3)	
40–49	151,955 (26.4)	41,940 (26.5)	41,506 (20.8)	
50–59	146,652 (25.5)	39,510 (24.9)	55,068 (27.6)	
60–69	113,450 (19.7)	32,227 (20.3)	60,721 (30.4)	
Pampalon Material Deprivation				< 0.0001
1 - Least Deprived	114,699 (19.9)	27,431 (17.3)	31,849 (15.9)	
2	112,179 (19.5)	28,171 (17.8)	32,274 (16.2)	
3	111,657 (19.4)	29,995 (18.9)	36,209 (18.1)	
4	113,430 (19.7)	32,929 (20.8)	42,935 (21.5)	
5 - Most Deprived	103,424 (18.0)	33,764 (21.3)	47,149 (23.6)	
Pampalon Social Deprivation				< 0.0001
1 - Least Deprived	111,614 (19.4)	26,802 (16.9)	30,300 (15.2)	
2	97,259 (16.9)	24,569 (15.5)	26,652 (13.3)	
3	108,872 (18.9)	29,293 (18.5)	34,559 (17.3)	
4	118,993 (20.7)	34,311 (21.7)	44,038 (22.1)	
5 - Most Deprived	118,651 (20.6)	37,315 (23.5)	54,867 (27.5)	
Geography				< 0.0001
Urban	470,513 (81.7)	123,369 (77.8)	150,384 (75.3)	
Rural	105,196 (18.3)	35,125 (22.2)	49,377 (24.7)	
Zone				< 0.0001
North	54,068 (9.4)	20,368 (12.9)	25,519 (12.8)	
Edmonton	190,709 (33.1)	49,626 (31.3)	60,840 (30.5)	
Central	59,052 (10.3)	18,464 (11.7)	27,028 (13.5)	
Calgary	234,602 (40.8)	59,402 (37.5)	69,818 (35.0)	
South	37,278 (6.5)	10,634 (6.7)	16,556 (8.3)	
GP visits in 3 years prior to index				< 0.0001
Yes	571,015 (99.2)	135,480 (85.5)	131,825 (66.0)	
No	4,695 (0.8)	23,014 (14.5)	67,936 (34.0)	
Continuity of Care (UPC)				< 0.0001
High Continuity	115,817 (20.1)	26,413 (16.7)	30,104 (15.1)	
Moderate Continuity	81,262 (14.1)	18,203 (11.5)	16,780 (8.4)	
Low Continuity	354,290 (61.5)	76,088 (48.0)	67,542 (33.8)	
No Continuity	24,341 (4.2)	37,790 (23.8)	85,335 (42.7)	
CIHI Health Profile Group				< 0.0001
Health System User, with health conditions	494,949 (86.0)	110,941 (70.0)	109,605 (54.9)	
Health System User, no health conditions	49,500 (8.6)	10,643 (6.7)	10,314 (5.2)	
Health System Non-user	31,261 (5.4)	36,908 (23.3)	79,823 (40.0)	
Driving distance to closest primary care clinic in minutes				< 0.0001
0 to 10 min	525,162 (91.2)	142,474 (89.9)	179,299 (89.8)	
11 to 20 min	25,889 (4.5)	7,593 (4.8)	9,176 (4.6)	
21 to 30 min	12,817 (2.2)	4,122 (2.6)	5,172 (2.6)	
> 30 min	11,745 (2.0)	4,213 (2.7)	5,977 (3.0)	

Table 2 presents the results of the multinomial logistic regression analyses examining factors associated with NRS and overdue for screening compared to CUTD. Those in older age groups were more likely to be associated with having NRS compared to those in younger age groups. Those 60–69 years of age were over 3 times more likely to have no record of screening compared to those 28–39 years of age (OR: 3.63; 95% CI: 3.57 to 3.70). There were also social and material deprivation gradients in odds of having NRS, where people living in more socially or materially deprived areas were more likely to have NRS compared to those living in less socially and materially deprived areas. A similar, but less steep, gradient was observed among odds of being overdue for screening. Regional differences were also present, those in the South Zone were over 1.5 times more likely to have NRS compared to those in Calgary Zone (OR: 1.51; 95% CI: 1.47 to 1.55). While those in the North Zone were over 1.36 times more likely to be overdue compared to those in Calgary Zone (OR: 1.36; 95% CI: 1.33 to 1.39). Those who were health system non-users were nearly 3 times more likely to have NRS compared to those who used the health system and had no health conditions (OR: 2.95: 95% CI: 2.86 to 3.04). This trend was also observed among those who were overdue for cervical cancer screening. Those with no GP visits in the 3 years prior to index date were over 13 times more likely to have NRS compared to those who visited a GP in the 3 years prior to index (OR: 13.86; 95% CI: 13.32 to 14.43). Those with no GP visits in the 3 years prior to index date were over 5 times more likely to be overdue compared to those who visited a family doctor in the 3 years prior to index (OR: 5.42; 95% CI: 5.20 to 5.65). Those with no usual provider of care were over 3 times more likely to have NRS compared to those with a high usual provider of care (i.e. consistently seeing the same physician across previous appointments) (OR: 3.227; 95% CI: 3.141 to 3.315). Similar results were found for those who are overdue. Finally, women who had to drive 30 min or more to their closest primary care centre were more likely to have NRS compared to those who were within 10 min driving distance to their closest primary care centre (OR: 1.10; 95% CI: 1.05 to 1.14). Model goodness of fit was assessed using the Hosmer–Lemeshow test and the Nagelkerke pseudo R². The Hosmer–Lemeshow test was statistically significant (*p* < 0.05) and The Nagelkerke pseudo R² value was 0.27. In multinomial regression, values between 0.2 and 0.4 are generally considered indicative of good model fit. It is important to note that a relatively low pseudo R² and statistically significant Hosmer–Lemeshow test does not necessarily imply a poor model. We retained the model in its current form. The model was meaningful as the variables are theoretically grounded, has clinically/public health relevance, and statistically significant.

**Table 2:** Unadjusted and adjusted multinomial logistic regression model examining the odds of NRS and overdue for cervical cancer screening compared to CUTD

Characteristic	Overdue	NRS	Overdue	NRS
	Unadjusted OR (95% CI)		Adjusted OR (95% CI)	
Age				
28–39	ref	ref	ref	ref
40–49	1.01 (0.99, 1.03)	1.05* (1.04, 1.07)	1.12* (1.10, 1.13)	1.31* (1.28, 1.33)
50–59	0.99 (0.97, 1.00)	1.45* (1.43, 1.47)	1.20* (1.18, 1.22)	2.23* (2.19, 2.27)
60–69	1.04* (1.02, 1.06)	2.07* (2.04, 2.10)	1.35* (1.32, 1.37)	3.63* (3.57, 3.70)
Pampalon Material Deprivation				
1 - Least Deprived	ref	ref	ref	ref
2	1.05* (1.03, 1.07)	1.04* (1.02, 1.05)	1.13* (1.11, 1.15)	1.22* (1.20, 1.25)
3	1.12* (1.10, 1.14)	1.17* (1.15, 1.19)	1.18* (1.15, 1.20)	1.30* (1.28, 1.33)
4	1.21* (1.19, 1.24)	1.36* (1.34, 1.39)	1.24* (1.22, 1.27)	1.46* (1.43, 1.49)
5 - Most Deprived	1.36* (1.34, 1.39)	1.64* (1.61, 1.67)	1.43* (1.41, 1.46)	1.86* (1.82, 1.89)
Pampalon Social Deprivation				
1 - Least Deprived	ref	ref	ref	Ref
2	1.06* (1.04, 1.08)	1.01 (0.99, 1.03)	1.05* (1.03, 1.08)	1.04* (1.02, 1.06)
3	1.12* (1.10, 1.14)	1.17* (1.15, 1.19)	1.10* (1.08, 1.12)	1.16* (1.13, 1.18)
4	1.20* (1.18, 1.23)	1.37* (1.35, 1.39)	1.14* (1.12, 1.16)	1.25* (1.23, 1.28)
5 - Most Deprived	1.31* (1.29, 1.34)	1.71* (1.68, 1.74)	1.18* (1.16, 1.20)	1.42* (1.40, 1.45)
Geography				
Urban	ref	ref	ref	ref
Rural	1.27* (1.25, 1.29)	1.47* (1.45, 1.49)	1.00 (0.98, 1.02)	1.04* (1.02, 1.07)
Zone				
Calgary	ref	ref	ref	ref
Central	1.24* (1.21, 1.26)	1.54* (1.51, 1.56)	1.18* (1.15, 1.21)	1.45* (1.42, 1.49)
Edmonton	1.02* (1.01, 1.04)	1.06* (1.05, 1.08)	1.05* (1.04, 1.07)	1.14* (1.12, 1.16)
North	1.48* (1.46, 1.51)	1.58* (1.55, 1.61)	1.36* (1.33, 1.39)	1.38* (1.35, 1.42)
South	1.13* (1.10, 1.15)	1.49* (1.45, 1.52)	1.11* (1.09, 1.14)	1.51* (1.47, 1.55)
GP visits in 3 years prior to index				
Yes	ref	ref	ref	ref
No	23.02* (22.24, 23.81)	69.77* (67.54, 72.07)	5.42* (5.20, 5.65)	13.86* (13.32, 14.43)
Continuity of Care (UPC)				
High Continuity	ref	ref	ref	ref
Moderate Continuity	0.98 (0.96, 1.00)	0.80* (0.78, 0.82)	1.00 (0.98, 1.02)	0.89* (0.87, 0.91)
Low Continuity	0.94* (0.93, 0.95)	0.74* (0.72, 0.75)	0.99 (0.97, 1.00)	0.86* (0.84, 0.87)
No Continuity	6.96* (6.81, 7.11)	13.82* (13.55, 14.01)	2.73* (2.66, 2.81)	3.23* (3.14, 3.32)
CIHI Health Profile Group				
Health System User, no health conditions	ref	ref	ref	ref
Health System Non-user	5.54* (5.39, 5.69)	12.25* (11.94, 12.57)	2.32* (2.26, 2.39)	2.95* (2.86, 3.04)
Health System User, with health conditions	1.04* (1.02, 1.07)	1.06* (1.04, 1.08)	1.16* (1.13, 1.18)	1.25* (1.22, 1.28)
Driving distance to closest primary care clinic in minutes				
0 to 10 min	ref	ref	ref	ref
11 to 20 min	1.07* (1.05, 1.10)	1.03 (1.00, 1.05)	1.06* (1.03, 1.09)	1.02 (0.99, 1.05)
21 to 30 min	1.19* (1.15, 1.23)	1.19* (1.15, 1.23)	1.06 (1.02, 1.10)	0.98 (0.94, 1.02)
> 30 min	1.34* (1.29, 1.40)	1.55* (1.50, 1.61)	1.07 (1.02, 1.11)	1.10* (1.05, 1.14)

*The Bonferroni correction was applied to correct for multiple comparisons. Accordingly, the Bonferroni-corrected, *p* < 0.001 is considered significant

**Fig. 3:**
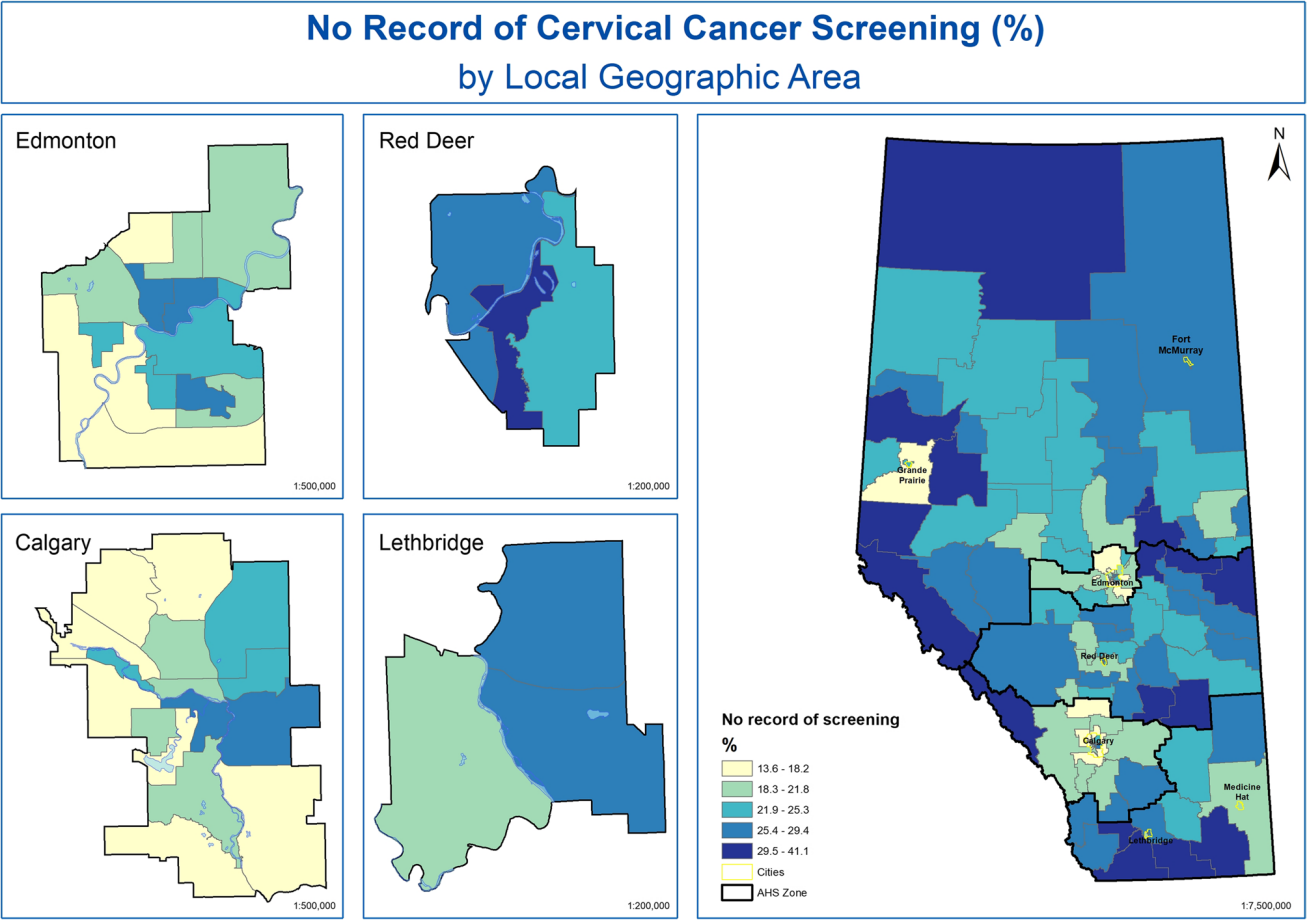
Percent of women with no record of cervical cancer screening by LGA in Alberta, Canada

**Fig. 4:**
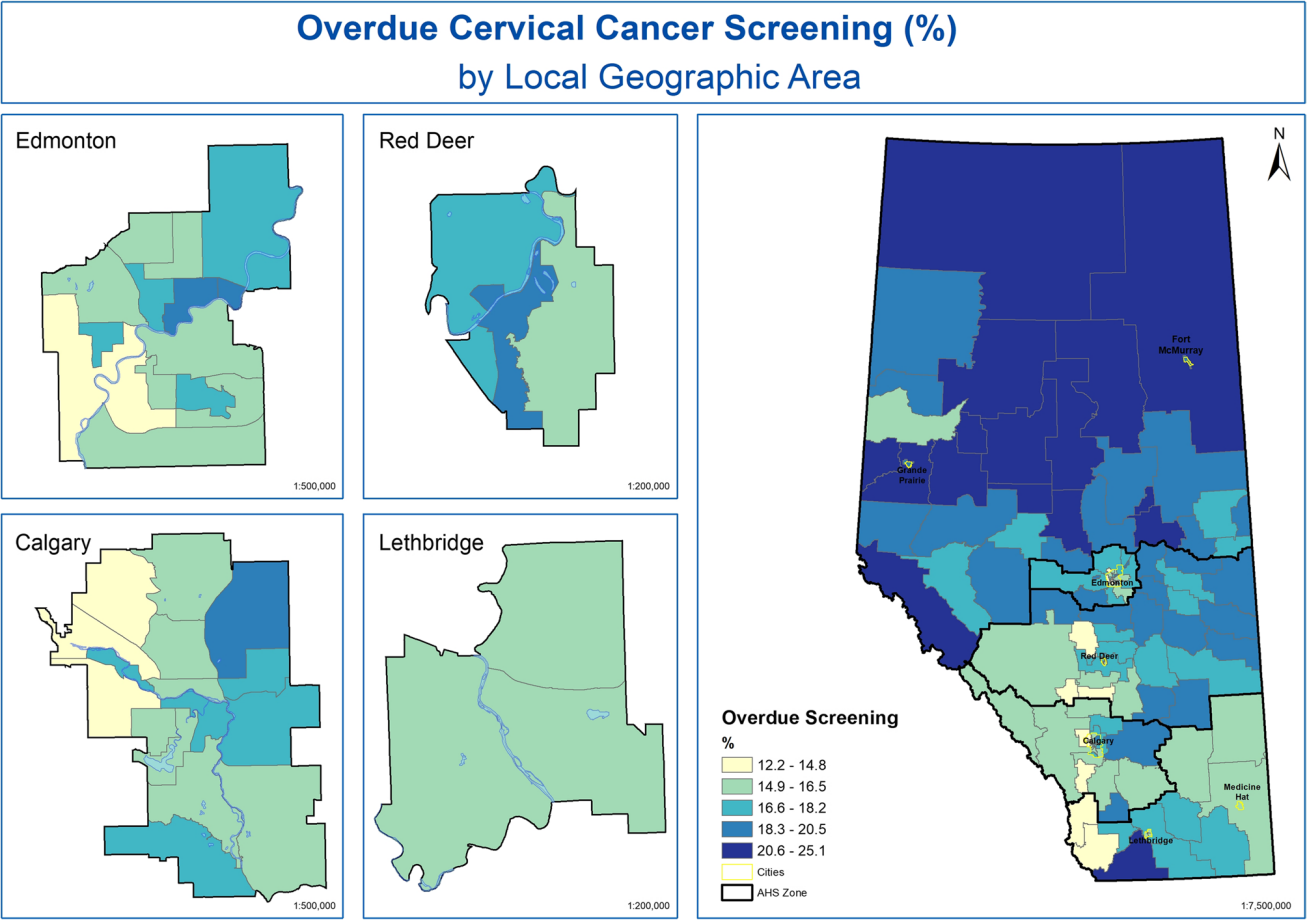
Percent of women overdue for cervical cancer screening by LGA in Alberta, Canada

Figure 3 and Fig. 4 present the percent of women with no record of cervical cancer screening and those overdue for cervical cancer screening by LGA in Alberta, Canada. Lower percentages are represented in light yellow and higher percentages are represented in dark blue. The percent of women with no record of cervical cancer screening ranged from the lowest 13.6% in Calgary-SE to the highest in Cardston-Kanai (41.1%). There are pockets of high proportion of no record of cervical cancer spread across all Alberta health zones.

The LGA with the lowest percent of women who are overdue for cervical cancer screening is in Crowsnest Pass (12.3%) and the highest in Wabasca (25.1%). Again, we see pockets of high proportion overdue for cervical cancer spread across all Alberta health zones but with particularly high proportions in the North and Central zones.

Results from the Global Moran’s I index indicate that the distribution of women with no record of cervical cancer screening and those overdue for cervical cancer screening are statistically significantly spatially autocorrelated (see Supplementary Material A). In other words, we reject the null hypothesis and conclude there is evidence for positive spatial autocorrelation (i.e., a clustered pattern) by LGA in Alberta, Canada. To identify the location and magnitude of these clusters we conducted a LISA and hot- and cold-spot analysis.

Findings from the Local Moran’s I (i.e., LISA) revealed localized clusters of similar values (high-high or low-low), and dissimilar ones (high-low or low-high). Specifically, for women with NRS, there are nine high-high local clusters: one in the North Zone, Spirit River LGA, one in the Central Zone, Wainwright LGA and remaining seven are in the southwest of South zone, two in City of Lethbridge. Additionally, low-low clusters are observed in and around the two metropolitan areas in Alberta, the City of Calgary and Edmonton, with high-low clusters present in and around both major cities (see Supplementary Material B). For women overdue for cervical screening, there are five high-high clusters, all located in the North Zone, predominantly in rural and remote areas, except for one urban area: the City of Grande Prairie. The metropolitan area of Calgary emerges as a low-low cluster, accompanied by several high-low clusters in the central and Calgary zones (see Supplementary Material C).

**Fig. 5:**
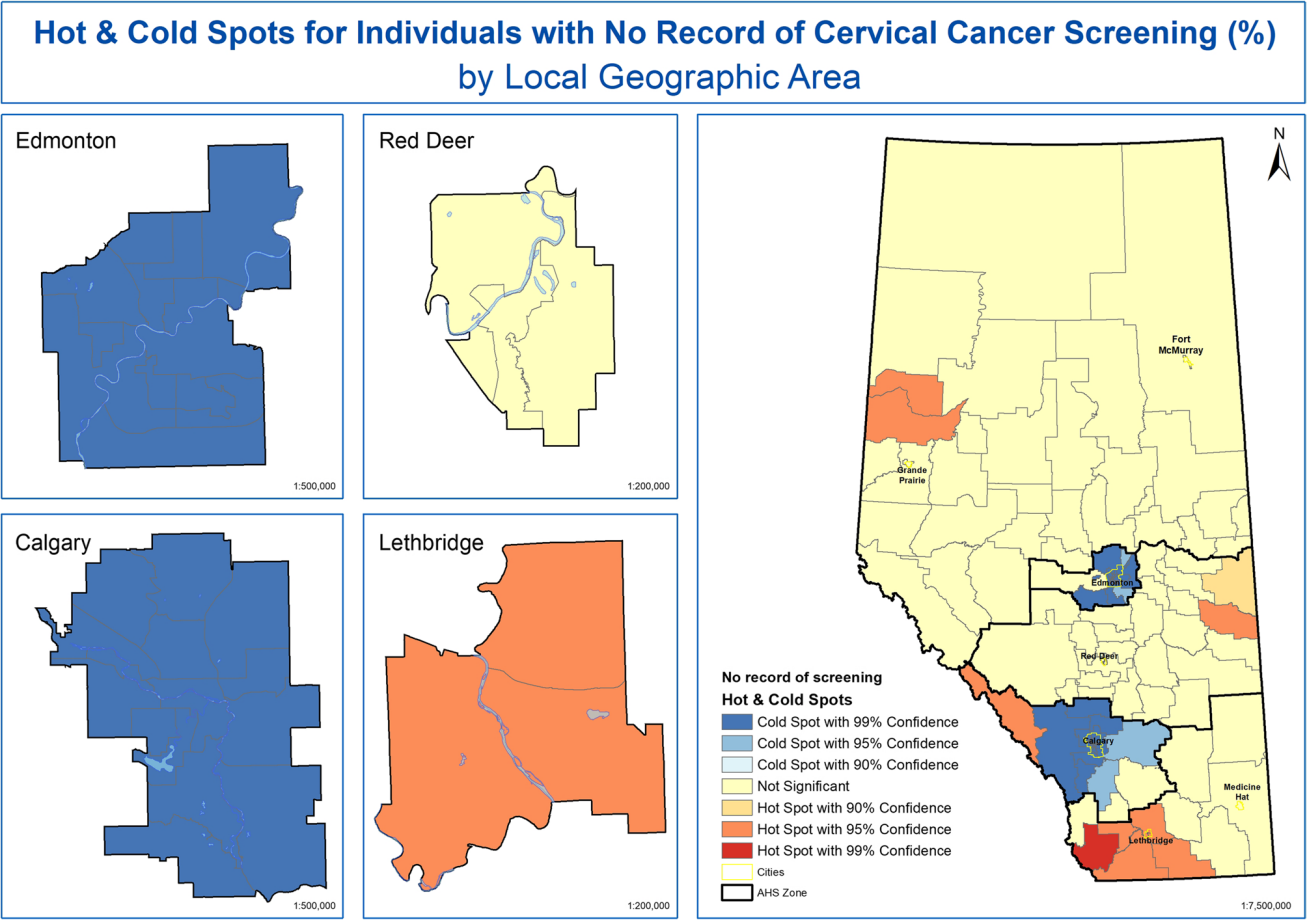
Hot- and Cold-spots for Women with NRS for Cervical Cancer by LGA in Alberta, Canada

Figure 5 visualizes the results from the optimized Getis-Ord Gi* hot spot analysis of women with NRS using an approximately 122 KM fixed threshold and 6 nearest neighbours as spatial weights. This resulted in 64 statistically significant LGAs identified as hot and cold spots. A statistically significant NRS cold spot means that LGAs with low proportion of women with NRS are surrounded by LGAs also with low proportion of women with NRS. A statistically significant hot spot means that LGAs with high proportion of women with NRS are surrounded by LGAs with high proportions. Results from Fig. 5 indicate that there are statistically significant hot spots of women who have no record of cervical cancer screening in the North Zone, the Central Zone, the Calgary Zone, and the South Zone. There are two statistically significant cold spots of women who have NRS, one in the Edmonton Zone and one in the Calgary Zone. Non-significant results (indicated by yellow) mean there is no evidence of women with NRS clustering in these LGAs.

**Fig. 6:**
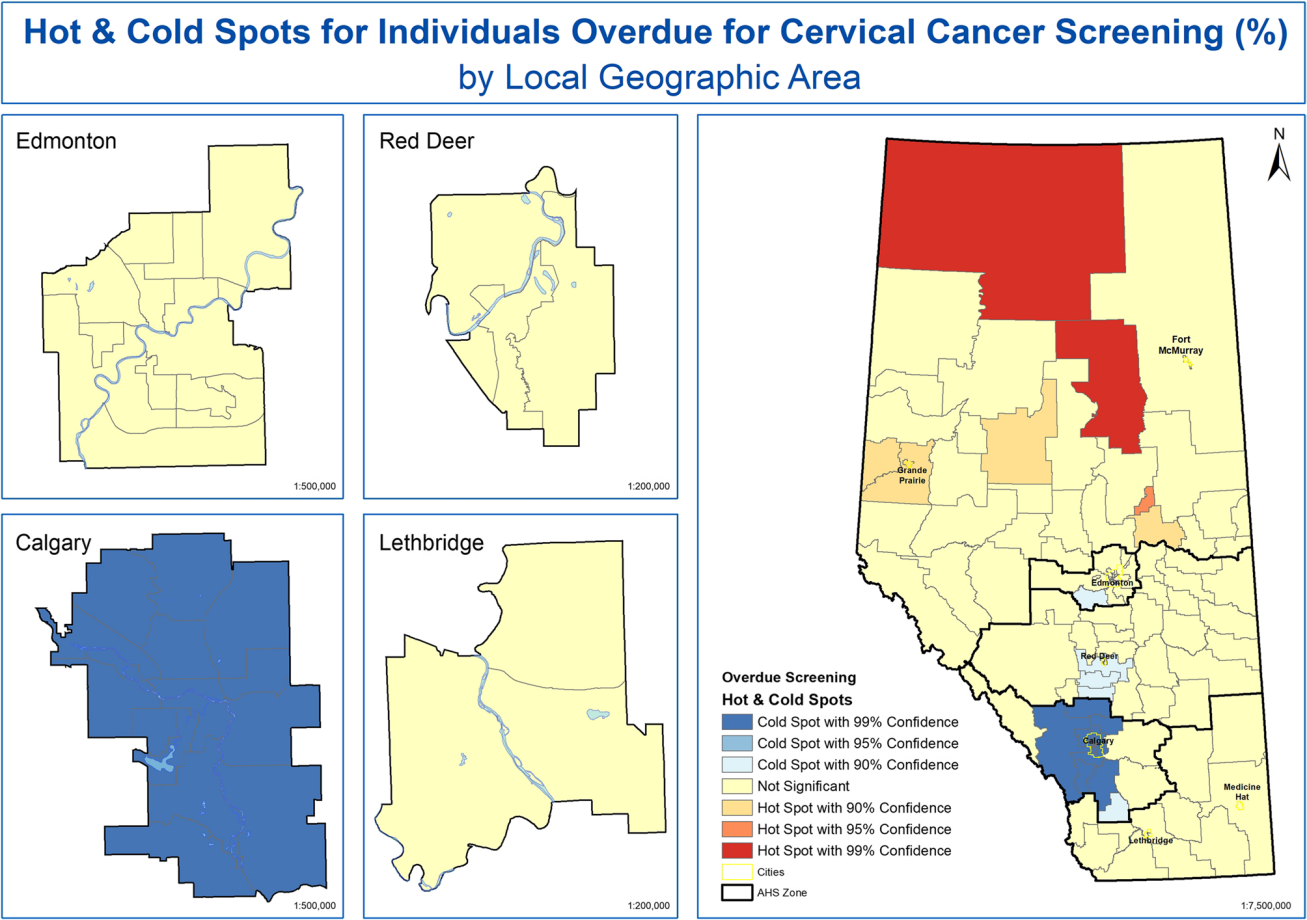
Hot- and Cold-spots for Women Overdue for Cervical Cancer Screening by LGA in Alberta, Canada

Figure 6 illustrates the results from the optimized Getis-Ord Gi* hot spot analysis of women overdue for cervical cancer screening using an approximately 122 KM fixed threshold and 6 nearest neighbours as spatial weights. This resulted in 38 statistically significant LGAs identified as hot and cold spots. Results for Fig. 6 indicate there are statistically significant hot spots in the North Zone. In addition, there is a small statistically significant cold spot in Edmonton Zone and a large statistically significant cold spot that crosses Central Zone and Calgary Zone. Non-significant results (indicated by yellow) mean there is no evidence of women overdue for cervical cancer screening clustering in these LGAs.

## Discussion

This population-based cross-sectional study identified and described the sociodemographic, health system, and geographical characteristics of eligible women’s cervical cancer screening participation. This study found that higher social and material deprivation, poorer access to healthcare providers, and living in rural locations have an impact on screening participation. These factors along with the specific LGAs in the South, Central, and North zones with lower cervical cancer screening identified in the geospatial analysis can be used to prioritize and plan public health initiatives to increase cervical cancer screening participation, especially among those who are underserved.

This study found that several health system utilization factors affected screening participation. Women who did not visit a family physician in the 3 years prior to the index date, those who did not use the healthcare system in the past year or had no usual provider of care in the 3 years prior to index were more likely to have NRS or be overdue for screening compared to those who did. In other studies, physician screening recommendations and endorsements are identified as facilitators to cancer screening uptake, so women who are able to visit a GP or those who use the health system are more likely to be up-to-date with screening [11, 34, 35].

Lack of access to health care can be a result of logistical barriers experienced by the individual, including lack of time, inability to take time off work, or difficulty with finding childcare or with scheduling [8–11]. Mobile screening can be a solution to bring additional health care into the community that can provide more flexibility in hours and locations to those who require logistical support. Mobile screening along with community outreach workers and patient navigators has been proposed as a promising intervention to reach individuals in the community who are experiencing difficulties interacting with the healthcare system [11, 36].

One potential intervention that could address some of these logistic barriers could be the provision of HPV self-sampling kits that can be used at home or in-clinic and have the potential to reach those who experience multiple barriers to screening and as a result have been under-screened [11]. HPV self-sampling involves the use of self-collected specimens to test for HPV and can be returned in-clinic or by mail. Using the at-home mail-in option address time and scheduling barriers where individuals may not be able to take time off work or those who have limited childcare options.

HPV self-sampling is increasingly being recommended and incorporated as a part of cervical cancer screening programs internationally, particularly in targeting underserved women [37]. In Ontario Canada, it has been shown that HPV self-sampling is perceived as a facilitator for cervical cancer screening that addresses these structural and logistical barriers in rural settings [38]. Moreover, as screening programs aim to recover in the wake of the COVID-19 pandemic, there has been a renewed focus on HPV self-sampling as a way of increasing screening rates and addressing inequities in screening that have widened due to the pandemic [6, 37, 39, 40]. Future research may wish to repeat this study in the years during and after the pandemic related distancing policies were lifted to determine the impact of the pandemic on geographic patterns of screening and health system and sociodemographic factors.

Additionally, the lack of health care access in certain areas is reflected in a lack of physicians, particularly in some rural and remote areas. Other types of primary care access including nurse practitioner service models and registered nurses who conduct screening exams may be especially relevant in such areas to increase cervical cancer screening participation. A recent review on interventions to target low breast, cervical, and colorectal cancer screening uptake in rural communities across the world found that multi-component nurse-led interventions that incorporate education and address structural barriers to screening were the most effective at increasing screening rates [41]. Interventions that address logistic barriers including mobile health clinics and HPV self-sampling can also help to address barriers to healthcare [11] access due to lack of health care providers and help to reduce geographic access issues for those living in rural or remote areas [11].

Interestingly, we observed that those with moderate or low continuity of care were less likely than those with high continuity of care to have no record of cervical cancer screening. This somewhat unexpected finding has previously been observed by Li et al. [42] who observed that increasingly continuity of care was associated with decreases in the odds of receiving cervical cancer screening. The authors hypothesized that this finding could be due to low knowledge of cervical cancer screening guidelines among providers and thus low adherence to cervical screening guidelines [43]. A pilot study conducted in clinics in Illinois found that providing healthcare providers with a multicomponent educational intervention increased adherence to guideline recommended cervical cancer screening [44]. Therefore, interventions focused on provider education on providing cervical cancer screening may reverse the relationship between continuity of care and cervical cancer screening over time.

Women who experience greater material or social deprivation were more likely to have NRS or be overdue for screening compared to those who experience less deprivation. Previous research conducted in Calgary, Alberta, found that the likelihood of cervical cancer screening decreased by 8% from least materially deprived to most materially deprivation, and by 10% for least socially deprived to most socially deprived demonstrating the impact of sociodemographic factors on screening outcomes [15]. Our study expands upon this work by extending this analysis to all of Alberta.

The findings of the present study are also aligned with past studies that have also shown that women living in socioeconomic deprivation or in areas of social deprivation have lower cervical cancer screening uptake [14, 45–47]. Material deprivation measures the deprivation of wealth, goods, as well as convenience. Therefore, solutions that can help to address convenience and indirect costs can impact material barriers to cervical cancer screening. Results of a systematic review have found that HPV self-sampling can improve cervical cancer screening participation among those in lower socioeconomic groups [48]. HPV self-sampling or mobile screening options offers more accessibility and convenience for those who may be underserved and can reduce the need for indirect costs like childcare if women can conduct self-sampling in-home.

Barriers to cancer screening also include social and cultural factors of inequality and family or community support and material factors such as literacy and education [8–12]. Providing avenues to improve knowledge of cervical cancer screening and offering culturally appropriate support (e.g. language and religious support) would help to address social and material barriers and likely improve cervical cancer screening participation [11, 49]. Theory-based educational interventions led by healthcare professionals that include cultural and linguistics components tailored to the community have been shown to be effective in increasing cervical cancer screening participation in the U.S [11]. and among rural communities internationally [50]. Recent engagements with underscreening individuals in Calgary, Alberta highlighted the need for an outreach strategy that contains multilingual video series to increase awareness and knowledge, social media promotion, and community education sessions that included a train the trainer model [51]. In addition, results from a recent meta-analysis have shown that patient navigators, which provide care coordinator, appointment reminders, and culturally tailored education, are an effective intervention for increasing screening rates for cervical, breast, and colorectal cancer among underserved populations (i.e., racial and ethnic minorities, people with lower SES, and people living in rural areas) [36].

Results from the multinomial logistic regression as well as the geospatial analysis indicate that there are areas in the North, Central, and South Zones that are hot spots for NRS. Women in these zones were also identified as being more likely to have NRS compared to those who live in the Calgary zone in the regression results. While hot-spot analysis and LISA serve different purposes [30, 31], it is intriguing to note that the two high-high cluster areas in women with NRS also align with hotspots in the South, Central and North zones. In the case of overdue screening, three high-high cluster areas also coincide with the hotspot map in the North zone.These areas warrant further investigation and present opportunities for targeted interventions to enhance screening participation in the long term. For example, many areas in the North, Central, and South Zones are considered rural or remote areas and it has been shown that those living in such settings may experience structural and logistical barriers to healthcare access, therefore addressing structural access issues may reduce or remove geographic inequities observed in this study [14, 52]. Furthermore, assessing interactions may be important in future studies to determine whether the observed associations between variables and the outcome depend on the context created by another variable (e.g., age). This deeper understanding could help identify specific subgroups that may require targeted attention or intervention.

### Strengths and Limitations

This study has several strengths. First, this study builds upon internal research done in Calgary and expands it by utilizing provincial population health administrative data for the entire province of Alberta [15]. Using administrative data allows for more accurate estimates of cervical cancer screening providing information at the individual level that provides higher level of validity. The use of administrative data also reduces response bias and recall bias that can be more an issue with studies that use survey data.

Second, sample sizes for each of the LGAs was sufficient to determine reliable estimates and no suppression of data was required thus allowing for a comprehensive understanding of the impact of the individual and geospatial characteristics on cervical cancer screening for all areas of the province. Third, this is the first Alberta study to combine the use of modelling and mapping techniques to allow for a more comprehensive understanding of cervical cancer screening in Alberta.

Lastly, this study was guided by a multi-disciplinary co-design team of experts in public health and cancer prevention and screening. Results from this study will be used to identify priority populations and areas in Alberta to target intervention planning and improve cervical cancer screening participation.

However, the study also has some limitations. First, there is a discrepancy between the time periods that data is being pulled for the number of GP visits variable and CIHI grouper variable. The time frame of the number of GP visits variable also affects the UPC indicator. The UPC variable uses the number GP visits in the 3 years prior to index date (2017–2019 calendar years) while the CIHI grouper variable looks at health care utilization in the 2019–2020 fiscal year. Thus, there are instances where an individual is considered a “health system non-user” from the CIHI grouper but have seen their GP regularly in 2017 and 2018 and thus misclassified as a “high UPC” in the usual provider of care variable. This occurred approximately among 7.9% of those considered a “health system non-user” in this data.

Second, the earliest available complete set of screening data from the ACCSP is January 1, 2012. Prior to 2012, ACCSP did not have provincial wide reach and thus only had data for 2 of the health regions in Alberta. Therefore, large proportions of people who may have been screened prior to this period, for example, if they moved from another province, will be classified as NRS. This could result in a misclassification of women as NRS when they are in fact overdue for screening.

Third, the use of the physician claims dataset to determine continuity of care and primary care visits limited our inclusion of other primary care providers like physicians on different compensation models (e.g. shadow billing or Alternative Relationship Plan (ARP)), nurse practitioners and other registered nurse services. This exclusion could underestimate the individual’s access to care. Fourth, the cervical screening database captures a small fraction of total hysterectomies in Alberta. Therefore, there are those included in the study who may not be eligible for cervical cancer screening but were misclassified as having NRS or overdue for screening.

Fifth, there was evidence of collinearity between the health system use variable and the number of visits to GP, this could result in an increase in the variance of these estimates. Sensitivity analysis was done to examine model results where usual provider of care was the only health utilization variable included in the model. Compared to the results in Table 2, results from the sensitivity analysis (supplementary material D) were similar in direction but had a difference in magnitude of the estimates. For example, those with no continuity of care were even more likely to have no record of screening.

Sixth, while we recognize that our use of the LGA level creates the potential for Modifiable Area Unit Problem (MAUP) [53] and ecological fallacy [54]. MAUP can be introduced through scale effect or the zoning effect. The scale effect arises from the varying sizes of spatial units. Smaller spatial units may reveal more localized patterns, while larger spatial units might generalize these patterns. The zoning effect occurs due to changes in aggregation based on different zoning configurations. However, the ethics approval was only obtained for utilizing data at the LGA level, therefore, all analyses were conducted and presented at this geographic scale. The analytical findings presented in this paper are only valid at the LGA scale and no inferences should be drawn at different scales. There is the potential for individual variation within each LGA and analyzing data at a lower level (such as neighborhood or sub-LGA) or a higher level (such as Sub-Zone or Zone) [19] may yield different results. However, the LGA has been used extensively in Alberta for health service planning and reporting since the establishment of AHS in 2009, making it the most appropriate level for potential interventions [26–28]. Finally, causal inferences cannot be drawn from this study due to its cross-sectional design and inherent data limitations. Although the model’s goodness-of-fit was relatively low, it includes variables that are theoretically grounded (with input from clinical and public health experts and based on literature), clinically relevant, and statistically significant. This study was designed to assess associations rather than to optimize predictive performance. Future research may consider developing a predictive model specifically aimed at accurately forecasting cervical cancer screening outcomes.

## Conclusion

This study contributes scientific knowledge that can guide public health initiatives, reducing disparities and enhancing cervical cancer prevention efforts in Alberta and potentially serve as a model for similar regions worldwide. To address disparities within cervical cancer rates we must be able to identify disparities in screening uptake. This study found that cervical cancer screening participation varied across geographical, health system and sociodemographic characteristics.

Health system access and utilization and social and material deprivation were key factors in determining cervical cancer screening participation. Interventions that address multiple factors of individual-level health care access and area-level deprivation would help to improve cervical cancer screening participation. Clusters of regions with higher proportions of women who are under-screened were identified in Alberta, Canada. These insights on the identified clusters and specific characteristics associated with under-screening can be used to provide public health practitioners with specific areas to target, tailor and pilot multi-level community-led interventions aimed at reducing inequities associated with lower cervical cancer screening uptake.

## Supplementary Information

Supplementary Material 1.

## Data Availability

The data that support the findings of this study are available from Alberta Health Services, but restrictions apply to the availability of these data, which were used under license for the current study, and so are not publicly available. Data are however available from the corresponding author upon reasonable request and with permission of Alberta Health Services.

## Abbreviations

ACCSP: Alberta Cervical Cancer Screening Program
AHCIP: Alberta Health Care Insurance Plan
AHS: Alberta Health Services
ARP: Alternative Relationship Plan
CIHI: Canadian Institutes of Health Information
CIHI POP Grouper: Canadian Institutes of Health Information Population Grouping Methodology
CI: Confidence Interval
CUTD: Currently Up-To-Date
DA: Dissemination Area
FDR: False Discovery Rate
GP: General Practitioner
HPV: Human Papillomavirus
LGA: Local Geographic Area
LISA: Local Indicator of Spatial Association
MAUP: Modifiable Areal Unit Problem
NACRS: National Ambulatory Care Reporting System
NRS: No Record of Screening
OR: Odds Ratios
Pap: Papanicolaou
PHN: Personal Healthcare Number
PCTF: Postal Code Translator File
SES: Socioeconomic Status
ULI: Unique Lifetime Identifier
UPC: Usual Provider of Care
WHO: World Health Organization
