# Supplementary material for: Individual-level characteristics and geospatial factors associated with cervical cancer screening participation in Alberta, Canada: a population-based cross-sectional study
Source: BMC Public Health. 2025 Aug 15;25:2790. doi: 10.1186/s12889-025-23898-4 (PMC12355785; doi:10.1186/s12889-025-23898-4)
Supplement: Supplementary file 1 — Supplementary Material 1. [file 12889_2025_23898_MOESM1_ESM.docx]

**Supplementary Materials**

**Supplementary Material A:** Results of Global Moran’s I Index of Spatial Autocorrelation

|  | **No Record of Cervical Cancer Screening** | **Overdue for Cervical Cancer Screening** |
| --- | --- | --- |
| Moran’s I Index | 0.422 | 0.264 |
| Z-Score | 7.55 | 4.78 |
| P-value | < 0.0001 | < 0.0001 |

**Supplementary B:** Local Spatial Autocorrelation for Individuals with No Record of Cervical Cancer Screening by Local Geographic Area


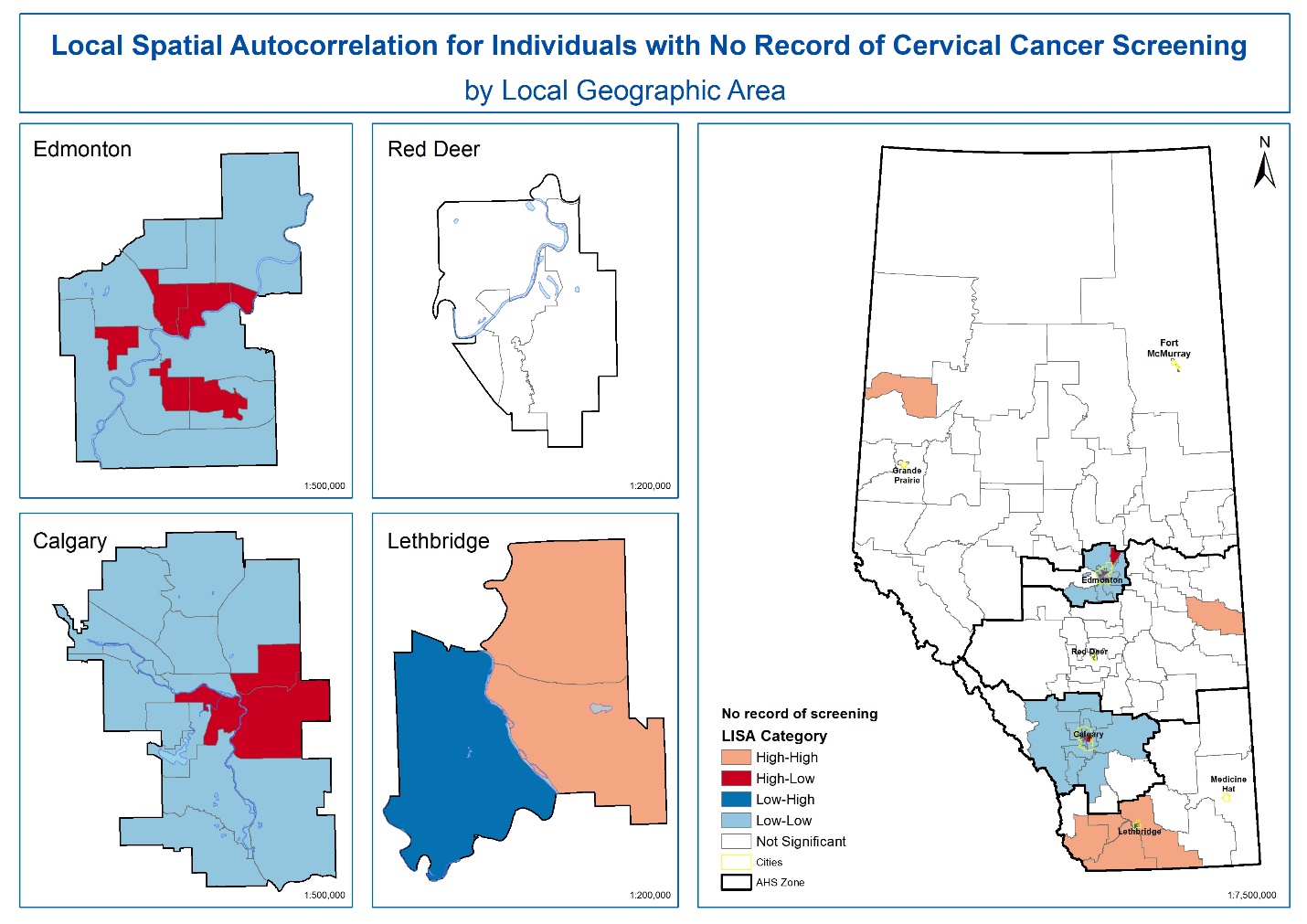


**Supplementary C:** Local Spatial Autocorrelation for Individuals Overdue for Cervical Cancer Screening by Local Geographic Area


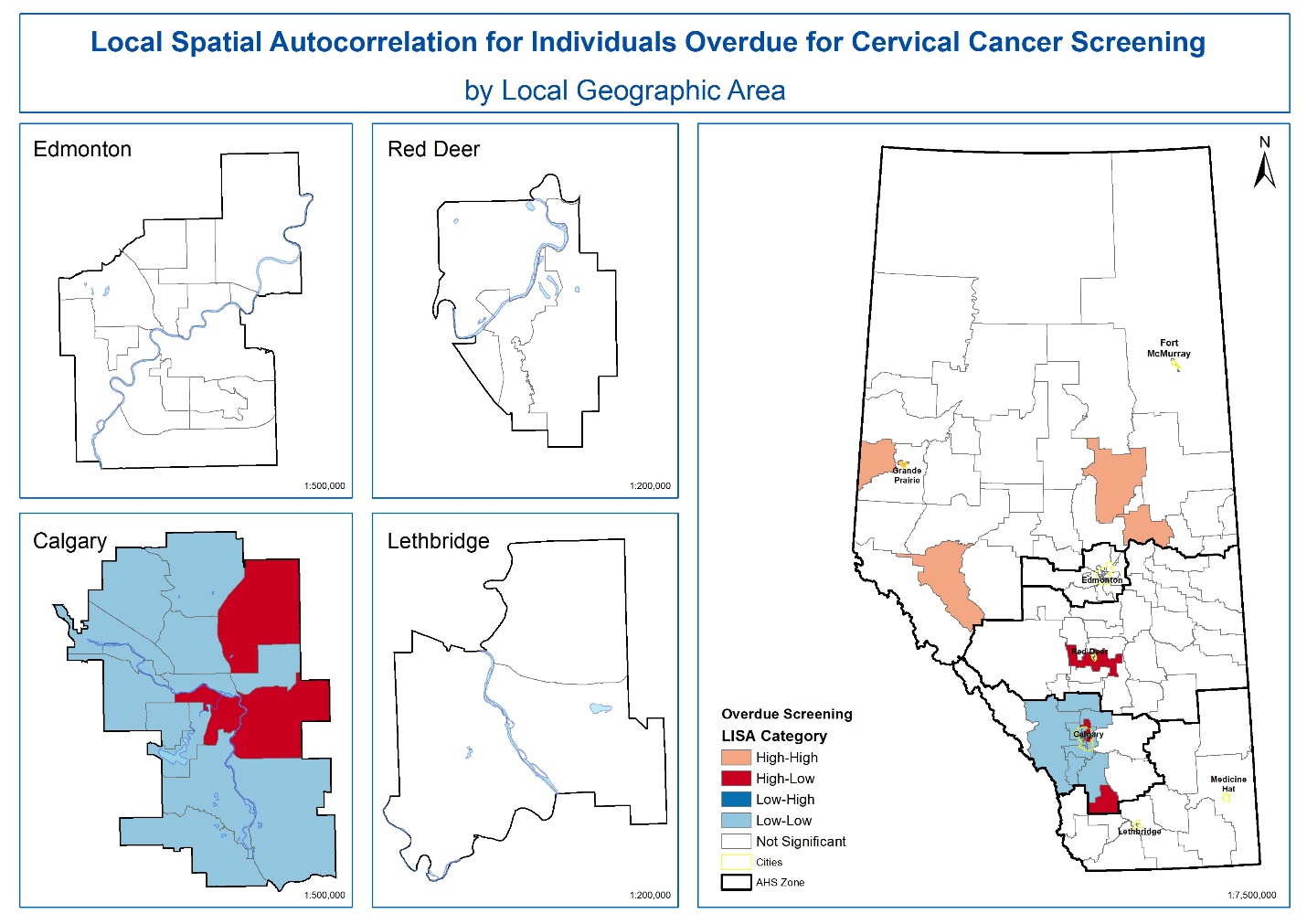


**Supplementary D:** Sensitivity Analysis. Adjusted multinomial logistic regression model examining the odds of NRS and overdue for cervical cancer screening compared to CUTD including only UPC as the health utilization variable

| **Characteristic** | **Overdue** | **NRS** |
| --- | --- | --- |
|  | **Adjusted OR**  **(95% CI)** | |
| **Age** |  |  |
| 28-39 | ref | ref |
| 40-49 | 1.09*  (1.07, 1.10) | 1.21*  (1.19, 1.23) |
| 50-59 | 1.15*  (1.13, 1.17) | 2.00*  (1.96, 2.03) |
| 60-69 | 1.29*  (1.26, 1.31) | 3.24*  (3.19, 3.30) |
| **Pampalon Material Deprivation** |  |  |
| 1 - Least Deprived | ref | ref |
| 2 | 1.12*  (1.09, 1.14) | 1.18*  (1.16, 1.21) |
| 3 | 1.16*  (1.14, 1.18) | 1.26*  (1.23, 1.28) |
| 4 | 1.23*  (1.20, 1.25) | 1.41*  (1.38, 1.43) |
| 5 - Most Deprived | 1.41*  (1.38, 1.44) | 1.77*  (1.74, 1.81) |
| **Pampalon Social Deprivation** |  |  |
| 1 - Least Deprived | ref | ref |
| 2 | 1.05*  (1.03, 1.08) | 1.04*  (1.02, 1.06) |
| 3 | 1.10*  (1.08, 1.12) | 1.16*  (1.14, 1.18) |
| 4 | 1.15*  (1.13, 1.17) | 1.27*  (1.25, 1.30) |
| 5 - Most Deprived | 1.20*  (1.18, 1.22) | 1.49*  (1.46, 1.52) |
| **Geography** |  |  |
| Urban | ref | ref |
| Rural | 1.00  (0.98, 1.02) | 1.01  (0.99, 1.03) |
| **Zone** |  |  |
| Calgary | ref | ref |
| Central | 1.17*  (1.14, 1.19) | 1.38*  (1.35, 1.41) |
| Edmonton | 1.04*  (1.02, 1.05) | 1.09*  (1.08, 1.11) |
| North | 1.34*  (1.31, 1.37) | 1.33*  (1.30, 1.36) |
| South | 1.10*  (1.07, 1.12) | 1.43*  (1.40, 1.47) |
| **Continuity of Care (UPC)** |  |  |
| High Continuity | ref | ref |
| Moderate Continuity | 1.00  (0.98, 1.02) | 0.89*  (0.87, 0.91) |
| Low Continuity | 0.97*  (0.95, 0.98) | 0.83*  (0.82, 0.85) |
| No Continuity | 7.40*  (7.24, 7.57) | 18.58*  (18.20, 18.97) |
| **Driving distance to closest primary care clinic in minutes** |  |  |
| 0 to 10 minutes | ref | ref |
| 11 to 20 minutes | 1.06*  (1.03, 1.09) | 0.99  (0.96, 1.02) |
| 21 to 30 minutes | 1.06  (1.02, 1.10) | 0.97  (0.93, 1.00) |
| >30 minutes | 1.07  (1.02, 1.11) | 1.09*  (1.05, 1.13) |

*Bonferroni-corrected p<0.0011 (α/44=0.05/44)

**Supplementary E:** Hosmer and Lemeshow test and Pseudo R² values (Nagelkerke R²) for model goodness of fit

| **Model Convergence Status** |
| --- |
| Convergence criterion (GCONV=1E-8) satisfied. |

| **R-Square** | 0.2314 | **Max-rescaled R-Square** | 0.2744 |
| --- | --- | --- | --- |

| **Hosmer and Lemeshow Goodness-of-Fit Test** | | |
| --- | --- | --- |
| **Chi-Square** | **DF** | **Pr > ChiSq** |
| 542.7599 | 16 | <.0001 |
